# Effect of Endoscopic Sinus Surgery on Eustachian Tube Function in Adult Sinusitis Patients: A Prospective Case-Control Study

**DOI:** 10.3390/jcm10204689

**Published:** 2021-10-13

**Authors:** Kyu Young Choi, Sookyung Jang, Ganghyeon Seo, Su-Kyoung Park

**Affiliations:** Department of Otorhinolaryngology-Head and Neck Surgery, College of Medicine, Kangnam Sacred Heart Hospital, Hallym University, Seoul 07441, Korea; coolq0@hallym.or.kr (K.Y.C.); pandapanda@hallym.or.kr (S.J.); steemedak@gmail.com (G.S.)

**Keywords:** eustachian tube, middle ear, sinusitis, tympanometry, Valsalva maneuver

## Abstract

The eustachian tube (E-tube) function is known to be related with sinusitis; however, the effect of endoscopic sinus surgery (ESS) on E-tube function is not clearly documented. This study aimed to prospectively evaluate the function of the E-tube by using both subjective and objective tests in adult chronic sinusitis patients undergoing ESS, and to compare with those of the patients without sinusitis. Thirty adult patients who underwent ESS for treatment of chronic sinusitis and another thirty patients without sinusitis who underwent other nasal surgeries (septoplasty, rhinoplasty, or closed reduction) were evaluated and compared for E-tube function before and after three months of their surgeries. The E-tube function tests included the seven-item eustachian tube dysfunction questionnaire (ETDQ-7), Valsalva test, and inflation-deflation test that were compared preoperatively and postoperatively in both groups. Compared with the group without sinusitis, the ESS group showed significant improvement of E-tube function after surgery in the ETDQ-7 (*p* = 0.002), right Valsalva test (*p* = 0.015), right deflation test (*p* = 0.005), and left deflation test (*p* = 0.006). A binary logistic regression analysis revealed that ESS significantly improved E-tube function in the right Valsalva test in a univariate (*p* = 0.021) and multivariate analysis (*p* = 0.008), and E-tube function in the left deflation test in a univariate (*p* = 0.021) and multivariate analysis (*p* = 0.039). These findings indicate that E-tube function is significantly improved after ESS in adult sinusitis patients, and that the presence of sinusitis and implementation of ESS should be considered (if sinusitis is present) in managing patients with ear diseases that are affected by E-tube function.

## 1. Introduction

The paranasal sinuses and nasal cavity are situated proximate with the middle ear, and connected to it by the eustachian tube (E-tube). So, diseases in these areas can affect each other, and it is not uncommon to meet patients in clinical practice with diseases in both nose and ear. Long standing paranasal sinusitis can cause swelling in the area of the E-tube orifice, which can result in deterioration of the E-tube function and consequently disease in the middle ear [1]. Direct flow of the postnasal drip in paranasal sinusitis into the E-tube can give rise to diseases in the E-tube or the middle ear [2,3]. Inflammatory responses of the mucosa and loss of the mucociliary transport function are also reported as the underlying pathomechanism of chronic sinusitis that lead to the deterioration of E-tube function [4,5].

Although E-tube dysfunction is well-known to be related to otologic diseases, the relationship of E-tube dysfunction with rhinologic diseases has not been reported much in adult patients compared with that in children. Marino et al. reported that the rhinologic patients demonstrated an increased burden of E-tube dysfunction symptoms that was correlated with increased sinonasal symptoms [1]. Stoikes and Dutton reported that E-tube dysfunction is common in patients with chronic sinusitis undergoing endoscopic sinus surgery (ESS), and that the symptoms resolved after the surgery [6]. Nevertheless, the reported studies on the relationship of sinusitis and E-tube function in adult patients are not based on the objective measurement of the E-tube function but questionnaires or analyses of previous studies. While E-tube dysfunction has been recognized as an important comorbidity associated with chronic sinusitis, a significant relationship between E-tube dysfunction and chronic sinusitis has clearly not been documented yet [1].

This study aimed to evaluate the E-tube functions of chronic sinusitis patients without ear symptoms or diseases who underwent ESS. The changes in E-tube function after treatment of chronic sinusitis were analyzed prospectively and compared with those without sinusitis. As no E-tube function test has been set yet for a standard diagnostic method of E-tube dysfunction [7,8,9], three objective tests using an impedance audiometer were used together in this study to directly measure the E-tube functions of the patients. The symptoms of the E-tube dysfunction were also assessed and compared using the eustachian tube dysfunction questionnaire (ETDQ-7) symptom questionnaire, which has been reported and acknowledged as a validated tool [2].

## 2. Materials and Methods

### 2.1. Subjects

From May 2018 to April 2019, 30 adult patients (older than 20 years of age) with chronic sinusitis undergoing ESS (ESS group) and 30 adult patients without chronic sinusitis (control group) undergoing other nasal surgeries (septoplasty, rhinoplasty, or closed reduction in nasal bone fracture) were prospectively recruited from a university hospital in Korea. All 60 patients had computed tomography scans taken of the paranasal sinuses before surgery, and the surgeries were performed by one experienced surgeon using similar techniques for each operation. Chronic sinusitis was diagnosed by the presence of more than three months of nasal symptoms and physical findings in nasal endoscopy or computed tomography. All the cases in the control group did not indicate symptoms of sinusitis and had no evidence of sinusitis in computed tomography scans or nasal endoscopy findings. ESS was performed for maxillary, frontal, ethmoid, and sphenoid sinuses according to the involved sites of sinusitis and polyps evaluated by the computed tomography scans. Antibiotics (mostly amoxicillin/clavulanic acid) were used for two weeks postoperatively and topical corticosteroids (mometasone furoate) were used for four weeks postoperatively in both groups. Exclusion criteria were patients with a previous operation, bleeding disorder, craniofacial anomaly, psychological problem, metabolic syndrome, other medical diseases, drug users, smokers, and heavy alcoholic drinkers (more than 30 g alcohol/day). Those with otologic diseases including otitis media, tinnitus, hearing loss, those without an intact tympanic membrane in the ear microscopy, and those without a clear middle ear or mastoid in computed tomography were also excluded to avoid other causes that can affect the function of the E-tube. Those who still showed evidence of sinusitis or polyp at the postoperative three-month follow-up were later excluded so that only those with completely treated sinusitis could be included in the study. To check the sinus-specific symptom and quality of life, the 22-item sino-nasal outcome test (SNOT-22) was used [10]. For the evaluation of chronic sinusitis, the Lund–Mackay (LM) computed tomography score and the Lund–Kennedy (LK) endoscopic scores were taken preoperatively [11].

The study was conducted according to the principles expressed in the Declaration of Helsinki, and was approved by the Institutional Review Board of Hallym University Kangnam Sacred Heart Hospital (IRB No. 2018-11-024) on 30 January 2019. Written informed consent was obtained from all patients.

### 2.2. Subjective Test

For the symptom questionnaire, a Korean version of the ETDQ-7 symptom questionnaire was used which is a translated version of the original ETDQ-7 symptom questionnaire [9]. It consisted of seven items of the symptoms (pressure in the ears?, pain in the ears?, a feeling that your ears are clogged or “under water”?, ear symptoms when you have a cold or sinusitis?, crackling or popping sounds in the ears?, ringing in the ears?, and a feeling that your hearing is muffled?), and patients were asked to score from 1 (no problem) to 7 (severe problem) for each item according to how much of those have been a problem for them over the past one month. Each patient took the symptom test twice, preoperatively one day before the surgery and postoperatively three months after the surgery. The total scores were compared between the two groups preoperatively, then they were classified as either “improved” (for a decreased total score compared with the preoperative score) or “not improved” (for the same or increased total score) postoperatively.

### 2.3. Objective Tests

The E-tube function of each patient was assessed by three objective tests: the Valsalva test, the inflation test, and the deflation test using GSI-TympStar impedance audiometer (Grason-Stadler Corp., Eden Prairie, MN, USA). For the Valsalva test, patients were asked to occlude the nostrils with their fingers and to blow air to the nose as in a Valsalva maneuver. An increased value was expected for pressure in normal E-tube function after the maneuver, and the test results were recorded as either positive (increased pressure) or negative (decreased pressure or no change) for E-tube function. Just as the symptom questionnaire, the objective tests had been performed one day before surgery preoperatively and three months after surgery postoperatively. They were further analyzed for improvement or no improvement of E-tube function after surgery. For the inflation-deflation test, a positive pressure of 200 mm H_2_O was applied first, and then the patients were asked to swallow repeatedly until no pressure change was noted and a plateau was reached. A decreased value was expected for pressure in the inflation test after swallowing for normal E-tube function, and the test results were recorded as either positive (reduced pressure) or negative (increased pressure or no change) for E-tube function. Then, the deflation test was conducted by applying a negative pressure of 200 mm H_2_O, while an increased value was expected after swallowing for normal E-tube function. The test results were recorded as either positive (increased pressure) or negative (decreased pressure or no change) for E-tube function in the deflation test. The results of the inflation-deflation tests were also performed preoperatively and postoperatively, then they were binarily classified into either “improved” or “not improved” groups after surgery for further analysis.

### 2.4. Statistical Analysis

A statistical analysis was performed using R language ver. 3.3.3 (R Foundation for Statistical Computing, Vienna, Austria). Data were expressed as a median (1st quartile–3rd quartile) for continuous variables. For categorical variables, data were expressed as a sample number and percentage (n, %). The Mann–Whitney U test was applied to compare the difference of a continuous response between the no sinusitis group and sinusitis group. The chi-squared test or Fisher’s exact test were performed to test the hypothesis of an association between sinusitis side and categorical responses using a contingency table. Univariate and multivariate analyses were performed to analyze the effect of sinusitis treatment on the improvement of E-tube function by binary logistic regression while adjusting the effect of age, sex, and sinusitis side. *p* values < 0.05 were considered statistically significant.

## 3. Results

### 3.1. Clinical Characteristics and Preoperative E-Tube Function

Because three patients in the ESS group still showed evidence of sinusitis or polyp in the physical examination after three months of surgery, they were then excluded and three other new patients whose sinusitis was completely dissolved three months after ESS were additionally included in the ESS group. Patients involved in this study were aged between 20 and 85 years of age with a mean age of 49.2 (±17.83) years of age (Table 1). Among the 60 cases, male patients were 42 and female patients were 18. In the ESS group, the sinusitis side was right in 8 cases and left in 10 cases, while the other 12 cases had sinusitis on both sides. Polyps were noted in 22 cases, fungal balls were found in 7 cases, sinusitis of dental origin was 2 cases, and no case was diagnosed with ciliary dyskinesia or mucoviscidosis. A total of 28 cases had sinusitis in the maxillary sinus, 14 had ethmoid sinusitis, 9 had frontal sinusitis, and 6 had sphenoid sinusitis. For the septoplasty group in control, septal deviation to the right side was noted in 12 cases while septal deviation to the left side was noted in the other 7 cases. The preoperative (baseline) E-tube function test values for both groups are presented in Table 2. The median preoperative ETDQ-7 symptom score was slightly higher in the ESS group compared with the control group, but they were not significantly different. The median seven individual item scores for the ESS group versus control groups were 1.57 (range, 1–4) vs. 1.67 (1–5) for “pressure in the ears?” (*p* = 0.709), 1.5 (1–4) vs. 1.43 (1–4) for “pain in the ears?” (*p* = 0.765), 2.33 (1–7) vs. 2.4 (1–7) for “a feeling that your ears are clogged or under water?” (*p* = 0.879), 2.73 (1–7) vs. 2.27 (1–7) for “ear symptoms when you have a cold or sinusitis?” (*p* = 0.311), 1.7 (1–7) vs. 1.2 (1–6) for “crackling or popping sounds in the ears?” (*p* = 0.022), 1.97 (1–6) vs. 1.63 (1–5) for “ringing in the ears?” (*p* = 0.325), and 2 (1–7) vs. 1.37 (1–4) for “a feeling that your hearing is muffled?” (*p* = 0.092). There were no significant correlations between the total ETDQ-7 score and SNOT-22 (*p* = 0.316), LM score (*p* = 0.467), or LK score (*p* = 0.694). The prevalence of negative E-tube function in preoperative Valsalva tests was noted in 63.3% for both sides in the ESS group, which was not significantly different from the control group. The preoperative inflation-deflation tests results were also not significantly different between the two groups.

### 3.2. Comparison of Postoperative E-Tube Function

The postoperative E-tube function test results are presented in Table 3. For the symptom score, the median total postoperative ETDQ-7 symptom score was 9.67 in the ESS group and 12.0 in the control group. When the cut-off value of 14.5 was used for the ETDQ-7 score [3,4], the number of cases with increased ETDQ-7 score reduced from 9 (30%) to 5 (16.7%) in the ESS group and 7 (23.3%) to 6 (20%) in the control group after surgery. The ratio of improved E-tube function (i.e., decreased score) after surgery was significantly higher in the ESS group compared with the control group (*p* = 0.002). The ESS group also showed a significantly higher ratio of improved E-tube function after surgery in the right Valsalva test, right deflation test, and left deflation test compared with the control group (*p* = 0.015, 0.005, 0.006, respectively). Higher ratios of improved E-tube function after surgery were also noted in the ESS group in the left Valsalva test, right inflation test, and left inflation test, but without statistical significance. For the 30 patients in the ESS group, the association analysis between the sinusitis side and the E-tube function of each side revealed an improvement in right Valsalva test after ESS in right sinusitis patients compared with both sinusitis patients (OR = 1.2) and an improvement in left Valsalva test after ESS in left sinusitis patients (OR = 1.6); however, no statistical significance was found (Table 4). The inflation test result showed no significant difference between each side of the sinusitis after treatment; however, the right deflation test showed significant improvement after ESS in right side sinusitis compared with both side sinusitis (OR = 2.917, *p* = 0.003).

### 3.3. Multivariate Regression Analysis for Sinusitis Treatment and Improvement of E-Tube Function

Binary logistic regression analyses were performed to analyze the effect of sinusitis treatment on the improvement of postoperative E-tube function while adjusting other factors such as sex, age, and sinusitis side (Table 5). The improvement of ETDQ-7 symptom score after treatment was significantly related to the treatment group (ESS group versus control group) in the univariate analysis (*p* = 0.002); however, no statistical significance was found after the multivariate analysis for factors sex, age, and sinusitis side (*p* = 0.058). Significant associations were noted between the treatment group and E-tube function improvement in the right Valsalva test or the left deflation test, for both the univariate and multivariate analyses. In the left inflation test and right deflation test, the surgical treatment of sinusitis improved E-tube function significantly in the univariate analysis; however, no statistical significance was found in multivariate analysis.

## 4. Discussion

Sinusitis is one of the most common diseases in otolaryngologic clinics, and its economic burden is significant [9]. Along with nasal symptoms, patients with sinusitis commonly present with comorbid chronic otitis media and E-tube dysfunction [9]. Because E-tube connects the middle ear and the nasal cavity, chronic inflammatory diseases are not only in the middle ear, sinuses and the nasal cavity can also affect E-tube function. Maniakas et al. found that patients with chronic sinusitis who fail maximal medical therapy and proceed to ESS have frequent E-tube dysfunctions [12]. They reported the symptom of ear fullness in close to two-thirds and ear pain in one-third of chronic sinusitis patients, and concluded that the E-tube dysfunction is greatly underappreciated in chronic sinusitis patients particularly with severe diseases [12].

There is much evidence that E-tube dysfunction occurs in patients with chronic sinusitis reported in the literature. The otologic symptom in chronic sinusitis patients was reported from 15 to 42% [6], and the presence of otitis media in adult chronic sinusitis patient was found in 23% [13]. Hong et al. found that the prevalence of chronic otitis media was significantly higher in subjects with chronic sinusitis in older patients with nasal polyps [14]. However, objective measurements of E-tube function in patients with chronic sinusitis is very limited [12], and it is not routinely performed for patients with paranasal sinusitis even though some present ear symptoms and it impacts patient quality-of-life [1]. Nevertheless, the relationship of sinusitis with E-tube dysfunction has been well-documented for pediatric patients. Due to the difference in anatomic nature and the function of the E-tube in children, many studies have reported a close relationship between chronic sinusitis and E-tube dysfunction in children [7,15]. The limited studies on E-tube dysfunction and chronic sinusitis are also attributable to the lack of appropriate diagnostic instruments for E-tube function tests. Since there is no specific E-tube function test in widespread clinical use [16], most clinicians are limited in daily practice for evaluating and managing patients with E-tube dysfunction. The diagnosis of E-tube dysfunction has been primarily based on the non-specific symptom or examination findings, rather than direct measurement of the E-tube function up to now [17]. Diverse diagnostic tests have tried to assess E-tube function including manometric assessments, sound transmission, electromyography, and imaging studies, etc. [3]. However, many of these tests are known to provide limited or selective information of E-tube function, and some of them are obtained in an expensive and complicated pressure chamber that is not readily available in clinical settings [18].

Tympanometry has been used traditionally to evaluate E-tube function, along with a swallow test or Valsalva test, etc. [19]. Tympanometry, an objective test, is a simple manometric test that can provide an indirect measure of E-tube function by measuring the pressure of the middle ear [8]. Using tympanometry, Doyle et al. reported that the Valsalva maneuver had sensitivity and specificity of 55% and 85% in discriminating between E-tube dysfunction and normal [20]. However, the pressure induced by the Valsalva maneuver is variable between patients. The sensitivity and specificity of the inflation test and deflation test on E-tube function were reported as 75% and 65%, and 73% and 58%, respectively [20]. The Valsalva, inflation, and deflation tests were proven to measure the E-tube function well in patients without otitis media by Swarts et al. [18].

Despite many attempts to develop objective E-tube function tests, no single reliable diagnostic tool has been reported as a gold standard [3,21,22]. In 2015, Smith and Tysome reported a systemic review of available E-tube function tests, which included 64 articles [16]. They concluded that no single test can be used as a gold standard, but combining different objective tests or adding patient-reported measures can improve accuracy for the diagnosis of E-tube dysfunction. By the combination of four manometric tests (Valsalva, E-tube opening pressure, inflation-deflation test), Doyle et al. reported an overall sensitivity and specificity of 95% and 83%, respectively, and concluded that the combination of the tests together can accurately identify ears with E-tube dysfunction with high sensitivity and specificity [20]. In this study, three different objective E-tube function tests (Valsalva, inflation, and deflation test) plus a symptom questionnaire were performed for all cases, and taken together to achieve a precise analysis of E-tube function.

While some studies have reported decreased E-tube function in sinusitis patients [2], most of them were based on an analysis of mere questionnaires or previous studies. None have directly measured the E-tube function in patients with sinusitis. Marino et al. reported that 43.3% of patients in rhinologic clinics had symptoms of E-tube dysfunction and that sinonasal symptoms correlated with E-tube dysfunction symptoms; however, their evaluation was only based on symptom scores [1]. Tangbumrungtham et al. reported a prevalence of 48.5% of E-tube dysfunction in chronic rhinosinusitis using ETDQ-7 [2].

E-tube function changes after treatment of chronic sinusitis have not been well-studied. The E-tube dysfunction symptom resolution in chronic sinusitis patients after ESS has been reported by Stoikes and Dutton [6]. They suggested the resolution of rhinosinusitis and disturbance of natural mucociliary clearance in the nasopharynx after ESS as the etiology of improved E-tube function; however, the study lacked objective E-tube function tests [6]. Improved obstruction and inflammation of the sinuses after ESS was also reported as one of the reasons for improved E-tube function after ESS [7]. Our results revealed significant improvement of E-tube function in sinusitis patients after complete treatment compared with control in both subjective and objective tests. To our knowledge, this study is the first to analyze the E-tube functions of chronic sinusitis patients with both objective and subjective studies, and prospectively analyze after surgical treatment, and compare with a control group.

The limitation of this study is that the possible impact on E-tube function after surgery of nasal septal deviation and the improvement of nasal breathing in the control group was not considered [23,24]. Other nasal surgeries were selected as a control group to compare with the ESS group; however, to avoid this, other otolaryngologic procedures outside the nose might have been good candidates for the control group. Using patients undergoing other types of nasal surgeries for control may have hindered the comparative strength, so future studies should consider using a control group free of any nasal or otologic surgical interventions and/or pathologies. The second limitation is that the subgroups of sinusitis (polyp presence or dental origin, etc.) or the extent of sinusitis (analyzing each specific sinuses) have not been evaluated for the effect on the E-tube function separately. The vidian nerve at the floor of the sphenoid sinus or the lesser palatine nerve passing the wall of the maxillary sinus, for example, can affect the levator palatini muscle influencing E-tube function or cause referred otalgia influencing ETDQ-7 [25,26]. The effect of medications including topical corticosteroids prescribed to the patients and the three excluded cases with persistent sinusitis after ESS also burden the possibility of bias in this study. Slightly right dependent (about 20°) head position during ESS might have influenced the different results of right and left E-tube function tests. Lastly, the correlation study between E-tube function tests was not performed. In the future, studies of the influence of each specific sinuses on E-tube function with larger sample sizes are needed.

Since E-tube dysfunction can lead to diverse otologic diseases [8,12,19], it is essential to address and manage the function of the E-tube in managing patients with ear diseases. The clinical significance of our study includes that the treatment of sinusitis improves E-tube dysfunction, so clinicians should always try to seek and manage possible sinusitis that will affect E-tube function and thus influence the otologic diseases. Although the E-tube dysfunction is not usually considered in managing sinusitis patients, concurrent or beforehand treatment of possible sinusitis is recommended in managing patients with ear diseases to improve the function of the E-tube.

## 5. Conclusions

Compared with the patients without sinusitis, significant improvement of E-tube function was noted after treatment of chronic sinusitis in subjective and some objective E-tube function tests, indicating the effect of sinusitis on E-tube function. In clinical practice, the patients with otologic diseases should be observed for concurrent chronic sinusitis and considered for ESS (if sinusitis is present), which can improve E-tube dysfunction after treatment of sinusitis.

## Figures and Tables

**Table 1:** Clinical characteristics of the patients.

Group	ESS Group	Control Group
Sex (male:female)	16:14	26:4
Age (mean ± SD)	52.8 ± 16.24	45.3 ± 19.71
SNOT-22 (mean ± SD)	39.2 ± 22.7	31.4 ± 19.1
LM score (mean ± SD)	18.1 ± 6.9	5.1 ± 4.7
LK score (mean ± SD)	6.7 ± 5.0	2.2 ± 2.0
Surgery		
ESS	30	0
Septoplasty	0	19
Rhinoplasty	0	6
Closed reduction	0	5

SD: standard deviation; ESS: endoscopic sinus surgery; SNOT-22: 22-item sino-nasal outcome test; LM score: Lund–Mackay computed tomography score; LK score: Lund–Kennedy endoscopic score.

**Table 2:** Baseline eustachian tube function test results of the patients.

E-Tube Function Test	Total (%)	ESS Group	Control Group	*p* Value
ETDQ-7 (median, range)	60 (100)	11 (7–17.5)	10.5 (7–15.75)	0.269
Right Valsalva test	60 (100)			0.194
Negative	33 (55)	19 (63.3)	14 (46.7)	
Positive	27 (45)	11 (36.7)	16 (53.3)	
Left Valsalva test	60 (100)			0.584
Negative	40 (66.7)	19 (63.3)	21 (70)	
Positive	20 (33.3)	11 (36.7)	9 (30)	
Right Inflation test	60 (100)			0.739
Negative	49 (81.7)	25 (83.3)	24 (80)	
Positive	11 (18.3)	5 (16.7)	6 (20)	
Left Inflation test	60 (100)			0.488
Negative	50 (83.3)	24 (80)	26 (86.7)	
Positive	10 (16.7)	6 (20)	4 (13.3)	
Right Deflation test	60 (100)			0.706
Negative	52 (86.7)	27 (90)	25 (83.3)	
Positive	8 (13.3)	3 (10)	5 (16.7)	
Left Deflation test	60 (100)			0.559
Negative	44 (73.3)	23 (76.7)	21 (70)	
Positive	16 (26.7)	7 (23.3)	9 (30)	

E-tube: eustachian tube; ESS: endoscopic sinus surgery; ETDQ-7: seven-item eustachian tube dysfunction questionnaire.

**Table 3:** Postoperative eustachian tube function test results of the patients.

E-Tube Function Test	Total (%)	ESS Group	Control Group	*p* Value
ETDQ-7	60 (100)			0.002 *
Not improved	36 (60)	12 (40)	24 (80)	
Improved	24 (40)	18 (60)	6 (20)	
Right Valsalva test	60 (100)			0.015 *
Not improved	46 (76.7)	19 (63.3)	27 (90)	
Improved	14 (23.3)	11 (36.7)	3 (10)	
Left Valsalva test	60 (100)			0.136
Not improved	45 (75)	20 (66.7)	25 (83.3)	
Improved	15 (25)	10 (33.3)	5 (16.7)	
Right Inflation test	60 (100)			0.117
Not improved	47 (78.3)	21 (70)	26 (86.7)	
Improved	13 (21.7)	9 (30)	4 (13.3)	
Left Inflation test	60 (100)			0.052
Not improved	52 (86.7)	23 (76.7)	29 (96.7)	
Improved	8 (13.3)	7 (23.3)	1 (3.3)	
Right Deflation test	60 (100)			0.005 *
Not improved	47 (78.3)	19 (63.3)	28 (93.3)	
Improved	13 (21.7)	11 (36.7)	2 (6.7)	
Left Deflation test	60 (100)			0.006 *
Not improved	50 (83.3)	21 (70)	29 (96.7)	
Improved	10 (16.7)	9 (30)	1 (3.3)	

E-tube: eustachian tube; ESS: endoscopic sinus surgery; ETDQ-7: seven-item eustachian tube dysfunction questionnaire. * *p* < 0.05.

**Table 4:** Association analysis between the sinusitis side and the E-tube function test results.

E-Tube Function Test	Total (%)	Not Improved	Improved	*p* Value	OR (95% CI)
ETDQ-7	30 (100)	12 (40)	18 (60)	0.156	
Both	13 (43.3)	4 (33.3)	9 (50)		1
Right	7 (23.3)	5 (41.7)	2 (11.1)		0.178 (0.024–1.339)
Left	10 (33.3)	3 (25)	7 (38.9)		1.037 (0.173–6.233)
Right Valsalva test	30 (100)	19 (63.3)	11 (36.7)	0.896	
Both	13 (43.3)	8 (42.1)	5 (45.5)		1
Right	7 (23.3)	4 (21.1)	3 (27.3)		1.2 (0.185–7.77)
Left	10 (33.3)	7 (36.8)	3 (27.3)		0.686 (0.119–3.963)
Left Valsalva test	30 (100)	20 (66.7)	10 (33.3)	0.071	
Both	13 (43.3)	8 (40)	5 (50)		1
Right	7 (23.3)	7 (35)	0 (0)		0.103 (0.005–2.191)
Left	10 (33.3)	5 (25)	5 (50)		1.6 (0.302–8.49)
Right Inflation test	30 (100)	21 (70)	9 (30)	0.276	
Both	13 (43.3)	10 (47.6)	3 (33.3)		1
Right	7 (23.3)	3 (14.3)	4 (44.4)		4.444 (0.616–32.07)
Left	10 (33.3)	8 (38.1)	2 (22.2)		0.833 (0.111–6.259)
Left Inflation test	30 (100)	23 (76.7)	7 (23.3)	1	
Both	13 (43.3)	10 (43.5)	3 (42.9)		1
Right	7 (23.3)	5 (21.7)	2 (28.6)		1.333 (0.165–10.743)
Left	10 (33.3)	8 (34.8)	2 (28.6)		0.833 (0.111–6.259)
Right Deflation test	30 (100)	19 (63.3)	11 (36.7)	0.003 *	
Both	13 (43.3)	7 (36.8)	6 (54.5)		1
Right	7 (23.3)	2 (10.5)	5 (45.5)		2.917 (0.407–20.9)
Left	10 (33.3)	10 (52.6)	0 (0)		0.055 (0.003–1.132)
Left Deflation test	30 (100)	21 (70)	9 (30)	0.184	
Both	13 (43.3)	7 (33.3)	6 (66.7)		1
Right	7 (23.3)	5 (23.8)	2 (22.2)		0.467 (0.065–3.344)
Left	10 (33.3)	9 (42.9)	1 (11.1)		0.13 (0.013–1.341)

E-tube: eustachian tube; OR: odds ratio; ETDQ-7: seven-item eustachian tube dysfunction questionnaire. * *p* < 0.05.

**Table 5:** Binary logistic regression analysis for factors influencing postoperative E-tube function.

E-Tube Function Test	Variable	Univariate OR (95% CI)	*p* Value	Multivariate OR (95% CI)	*p* Value
ETDQ-7	Treatment group	6 (1.89–19.043)	0.002 *	4.831 (0.949–24.578)	0.058
	Sex	1.8 (0.588–5.511)	0.303	0.762 (0.176–3.302)	0.717
	Age	1.054 (1.016–1.093)	0.005	1.046 (1–1.094)	0.048
	Sinusitis side	0.6 (0.107–3.352)	0.561	0.143 (0.018–1.136)	0.066
Right Valsalva test	Treatment group	5.211 (1.279–21.235)	0.021 *	20.023 (2.211–181.356)	0.008 *
	Sex	2.125 (0.611–7.391)	0.236	1.756 (0.384–8.022)	0.468
	Age	0.992 (0.959–1.026)	0.632	0.944 (0.896–0.994)	0.028
	Sinusitis side	1.4 (0.195–10.032)	0.738	2.768 (0.293–26.182)	0.374
Left Valsalva test	Treatment group	2.5 (0.735–8.502)	0.142	2.334 (0.444–12.267)	0.317
	Sex	0.805 (0.218–2.975)	0.745	0.408 (0.085–1.97)	0.264
	Age	1.005 (0.972–1.039)	0.787	0.994 (0.954–1.036)	0.783
	Sinusitis side	4 (0.967–16.551)	0.056	3.699 (0.658–20.796)	0.138
Right Inflation test	Treatment group	2.786 (0.751–10.332)	0.126	3.081 (0.509–18.667)	0.221
	Sex	1.048 (0.276–3.975)	0.945	0.672 (0.147–3.076)	0.609
	Age	1.009 (0.974–1.046)	0.603	0.989 (0.946–1.035)	0.642
	Sinusitis side	4 (0.5–31.982)	0.191	4.676 (0.547–40.002)	0.159
Left Inflation test	Treatment group	8.826 (1.012–76.963)	0.049 *	7.278 (0.577–91.826)	0.125
	Sex	2.714 (0.596–12.352)	0.196	1.47 (0.289–7.476)	0.602
	Age	1.033 (0.986–1.082)	0.169	1.007 (0.954–1.062)	0.839
	Sinusitis side	0.75 (0.081–6.958)	0.8	0.612 (0.062–5.988)	0.673
Right Deflation test	Treatment group	8.105 (1.611–40.767)	0.011 *	4.995 (0.79–31.595)	0.087
	Sex	1.048 (0.276–3.975)	0.945	0.365 (0.072–1.858)	0.225
	Age	1.047 (1.004–1.091)	0.031	1.029 (0.977–1.083)	0.28
	Sinusitis side	9.167 (1.819–46.205)	0.007 *	6.338 (0.971–41.359)	0.054
Left Deflation test	Treatment group	12.429 (1.461–105.741)	0.021 *	12.598 (1.142–138.952)	0.039 *
	Sex	1.714 (0.419–7.006)	0.453	0.95 (0.167–5.417)	0.954
	Age	1.065 (1.012–1.121)	0.015	1.049 (0.984–1.117)	0.141
	Sinusitis side	0.333 (0.024–4.548)	0.41	0.292 (0.018–4.703)	0.386

E-tube: eustachian tube; OR: odds ratio; ETDQ-7: seven-item eustachian tube dysfunction questionnaire. * *p* < 0.05.

## Data Availability

The data presented in this study are available on request from the corresponding author.

## References

[1] Marino M.J., Ling L.C., Yao W.C., Luong A., Citardi M.J. (2017). Eustachian tube dysfunction symptoms in patients treated in a tertiary rhinology clinic. Int. Forum Allergy Rhinol..

[2] Tangbumrungtham N., Patel V.S., Thamboo A., Patel Z.M., Nayak J.V., Ma Y., Choby G., Hwang P.H. (2018). The prevalence of Eustachian tube dysfunction symptoms in patients with chronic rhinosinusitis. Int. Forum Allergy Rhinol..

[3] McCoul E.D., Anand V.K., Christos P.J. (2012). Validating the clinical assessment of eustachian tube dysfunction: The Eustachian Tube Dysfunction Questionnaire (ETDQ-7). Laryngoscope.

[4] Van Roeyen S., Van de Heyning P., Van Rompaey V. (2015). Value and discriminative power of the seven-item Eustachian Tube Dysfunction Questionnaire. Laryngoscope.

[5] Yeo S.G., Park D.C., Eun Y.G., Cha C.I. (2007). The role of allergic rhinitis in the development of otitis media with effusion: Effect on eustachian tube function. Am. J. Otolaryngol..

[6] Stoikes N.F., Dutton J.M. (2005). The effect of endoscopic sinus surgery on symptoms of eustachian tube dysfunction. Am. J. Rhinol..

[7] Leo G., Piacentini E., Incorvaia C., Consonni D. (2007). Sinusitis and Eustachian tube dysfunction in children. Pediatr. Allergy Immunol..

[8] Makibara R.R., Fukunaga J.Y., Gil D. (2010). Função da tuba auditiva em adultos com membrana timpânica íntegra. Braz. J. Otorhinolaryngol..

[9] Ray N.F., Baraniuk J.N., Thamer M., Rinehart C.S., Gergen P.J., Kaliner M., Josephs S., Pung Y.H. (1999). Healthcare expenditures for sinusitis in 1996: Contributions of asthma, rhinitis, and other airway disorders. J. Allergy Clin. Immunol..

[10] Hopkins C., Gillett S., Slack R., Lund V.J., Browne J.P. (2009). Psychometric validity of the 22-item Sinonasal Outcome Test. Clin. Otolaryngol..

[11] Lund V.J., Mackay I.S. (1993). Staging in rhinosinusitus. Rhinology.

[12] Maniakas A., Desrosiers M., Asmar M.-H., Al Falasi M., Mfuna Endam L., Hopkins C., Philpott C., Erskine S., Smith R., Kilty S. (2018). Eustachian tube symptoms are frequent in chronic rhinosinusitis and respond well to endoscopic sinus surgery. Rhinology.

[13] Finkelstein Y., Talmi Y.P., Rubel Y., Bar-Ziv J., Zohar Y. (1989). Otitis media with effusion as a presenting symptom of chronic sinusitis. J. Laryngol. Otol..

[14] Hong S.-N., Lee W.H., Lee S.H., Rhee C.-S., Lee C.H., Kim J.-W. (2017). Chronic rhinosinusitis with nasal polyps is associated with chronic otitis media in the elderly. Eur. Arch. Oto-Rhino-Laryngol..

[15] Hong C.K., Park D.C., Kim S.W., Cha C.I., Cha S.-H., Yeo S.G. (2008). Effect of paranasal sinusitis on the development of otitis media with effusion: Influence of eustachian tube function and adenoid immunity. Int. J. Pediatr. Otorhinolaryngol..

[16] Smith M.E., Tysome J.R. (2015). Tests of Eustachian tube function: A review. Clin. Otolaryngol..

[17] Smith M.E., Takwoingi Y., Deeks J., Alper C., Bance M.L., Bhutta M.F., Donnelly N., Poe D., Tysome J.R. (2018). Eustachian tube dysfunction: A diagnostic accuracy study and proposed diagnostic pathway. PLoS ONE.

[18] Swarts J.D., Alper C.M., Mandel E.M., Villardo R., Doyle W.J. (2011). Eustachian tube function in adults without middle ear disease. Ann. Otol. Rhinol. Laryngol..

[19] Han W.G., Yoo J., Rah Y.C., Chang J., Im G.J., Song J.-J., Chae S.W., Jung H.H., Choi J. (2017). Analysis of Eustachian Tube Dysfunction by Dynamic Slow Motion Video Endoscopy and Eustachian Tube Dysfunction Questionnaire in Chronic Otitis Media. Clin. Exp. Otorhinolaryngol..

[20] Doyle W.J., Swarts J.D., Banks J., Casselbrant M.L., Mandel E.M., Alper C.M. (2013). Sensitivity and specificity of eustachian tube function tests in adults. JAMA Otolaryngol. Head Neck Surg..

[21] Llewellyn A., Norman G., Harden M., Coatesworth A., Kimberling D., Schilder A., McDaid C. (2014). Interventions for adult Eustachian tube dysfunction: A systematic review. Health Technol. Assess..

[22] Todd N.W. (2000). There are no accurate tests for eustachian tube function. Arch. Otolaryngol. Head Neck Surg..

[23] Nanda M.S., Kaur M., Bhatia S. (2017). Impact of Septoplasty on hearing and middle ear function. Int. J. Res. Med. Sci..

[24] DoĞAn R. (2019). The Effect of Types of Nasal Septum Deviation on the Eustachian Tube Function. Bezmialem Sci..

[25] Kishimoto H., Matsuura Y., Kawai K., Yamada S., Suzuki S. (2016). The Lesser Palatine Nerve Innervates the Levator Veli Palatini Muscle. Plast. Reconstr. Surg. Glob. Open.

[26] Senger M., Stoffels H.-J., Angelov D.N. (2014). Topography, syntopy and morphology of the human otic ganglion: A cadaver study. Ann. Anat.-Anat. Anz..

